# Markov State Models with Weighted Ensemble Simulation: How to Eliminate the Trajectory Merging Bias

**DOI:** 10.1021/acs.jctc.4c01141

**Published:** 2025-02-11

**Authors:** Samik Bose, Ceren Kilinc, Alex Dickson

**Affiliations:** † Department of Biochemistry and Molecular Biology, Michigan State University, East Lansing, Michigan 48824, United States; ‡ Department of Computational Mathematics, Science and Engineering, Michigan State University, East Lansing, Michigan 48824, United States

## Abstract

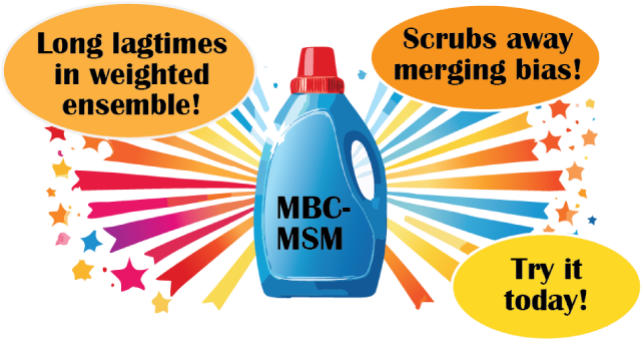

The weighted ensemble (WE) algorithm is gaining popularity as a rare event method for studying long timescale processes with molecular dynamics. WE is particularly useful for determining kinetic properties, such as rates of protein (un)folding and ligand (un)binding, where transition rates can be calculated from the flux of trajectories into a target basin of interest. However, this flux depends exponentially on the number of splitting events that a given trajectory experiences before reaching the target state and can vary by orders of magnitude between WE replicates. Markov state models (MSMs) are helpful tools to aggregate information across multiple WE simulations and have previously been shown to provide more accurate transition rates than WE alone. Discrete-time MSMs are models that coarsely describe the evolution of the system from one discrete state to the next using a discrete lag time, τ. When an MSM is built using conventional MD data, longer values of τ typically provide more accurate results. Combining WE simulations with Markov state modeling presents some additional challenges, especially when using a value of τ that exceeds the lag time between resampling steps in the WE algorithm, τ_WE_. Here, we identify a source of bias that occurs when τ > τ_WE_, which we refer to as “merging bias”. We also propose an algorithm to eliminate the merging bias, which results in merging bias-corrected MSMs, or “MBC-MSMs”. Using a simple model system, as well as a complex biomolecular example, we show that MBC-MSMs significantly outperform both τ = τ_WE_ MSMs and uncorrected MSMs at longer lag times.

## Introduction

Markov state models (MSMs) are powerful computational tools that have been used to analyze the conformational dynamics of biomolecules.^1−8^ An MSM is a kinetic model that describes the evolution of a system over a period of time, known as lag time, in terms of transitions between discrete states. For molecular dynamics (MD) simulations, a common approach is to use states that represent sets of conformations obtained by clustering the atomic positions or some salient features. The states and the transitions between them are assumed to follow Markovian dynamics: the future behavior of the system depends on only its current state and not on the path by which it arrived there. Transition probabilities between states can be calculated from MD simulation data and together form the transition probability matrix, which encodes the kinetics of the entire system.^6−8^ This enables MSMs to study slow processes and estimate relevant properties like transition rates with higher statistical significance than a stand-alone MD simulation. The consistent development of MSM methodology and software such as MSMBuilder, PyEMMA, and Deeptime has helped us bridge the gap between microscopic information and the macroscopic kinetic and thermodynamic observables of biomolecular systems such as protein (un)folding and ligand (un)binding.^9−11^ Recent advances in machine learning-based MSM frameworks such as VAMPNets have amplified the robustness of generating Markov models for studying such long timescale processes.^12−15^

In order to utilize MSMs to study the transition paths and rates, one must have a set of trajectories that connect the basins of interest. These can be challenging to generate, as these basins are often separated by large energy barriers that can only be crossed on extremely long timescales. Over the past decade, we have seen a remarkable advancement in the limits of MD simulations either by developing special-purpose supercomputers such as Anton or by implementing community-driven consortium such as Folding@Home.^16−18^ In the absence of specialized resources, many computational strategies have been developed for generating transition paths of long timescale events, which are collectively referred to as “enhanced sampling methods”.^19−21^ These methods can simulate molecular processes on timescales of seconds to hours or more, which are millions of times longer than the duration of a typical MD trajectory. Although countless new transition pathways have been discovered with enhanced sampling methods, acquiring high statistical significance of the pathways and related transition rates remains a major challenge.^22−24^ This is due to the fact that even the state-of-the-art enhanced sampling methods can only sample long timescale processes for a limited number of instances, and tight convergence of sampling can hardly be guaranteed within a limited computational time.^25^ Hence, building MSMs with enhanced sampling simulation data can lead to an efficient avenue to estimate the transition rates of processes on the order of seconds to hours.

Some widely used enhanced sampling methods such as various flavors of metadynamics and steered MD use biasing potentials to ensure the system samples higher free energy configurations.^26−30^ In order to build MSMs for a more robust prediction of transition rates from such enhanced sampling data, one must remove the underlying bias on the state-to-state transitions, which is not straightforward. In contrast, weighted ensemble (WE)-based enhanced sampling generates rare events using statistical enrichment strategies applied to an ensemble of trajectories.^31,32^ Each trajectory is assigned a weight which keeps track of the probability of such trajectories occurring naturally. These can be more easily combined with MSMs, as each trajectory is generated using the same unbiased Hamiltonian energy function.^33,34^ It carries out cloning and merging of statistically weighted trajectories to explore the low-probability regions of the configuration space. The WE strategy is based on nonequilibrium statistical mechanics and provides unbiased estimate of kinetics and sampling of equilibrium or nonequilibrium processes.^35,36^ As a limited number of uncorrelated rare event observations are generated in a given WE simulation, one major goal has always been to improve the statistical significance of the transition rates obtained. Hence, integrating MSMs with the WE framework is an obvious avenue for better understanding of long timescale events.

A unique advantage of the WE formalism is that one can use the statistical weights of WE trajectories to populate the time-lagged transition count matrices. Previously, there have been significant efforts toward building Markov models with WE data and subsequent estimation of transition pathways, rates, and committor probabilities.^37−41^ Adhikari and coworkers developed history-augmented MSMs (haMSM) with WE simulation data to characterize protein folding times in the order of seconds.^37^ Later, Copperman and Zuckerman implemented haMSMs within the WE framework to accelerate the converged estimation of protein folding kinetics.^42^ Previous results from our lab have utilized the simulation data from two different weighted ensemble algorithms, WExplore and REVO (resampling of ensembles by variation optimization), and have built MSMs to study long-timescale ligand (un)binding kinetics and mechanisms in several biomolecular systems.^25,39−41^ It has been demonstrated that the prediction of transition rates has improved substantially with the WE-MSM approach compared to direct estimates from WE simulation by Hill’s relation.^41^ To our knowledge, the lag times of the MSMs built with WE simulation data have always been restricted to one resampling cycle, i.e., the time between two consecutive merging/cloning cycles within the trajectory.^37,39,41^ This is typically of the order of 5–30 ps for standard WE-MSMs built thus far. On the other hand, it has been well documented for straightforward MD that longer lag times can help to identify the slowest processes and their underlying dynamics.^8^ In the context of WE-MSM, the use of longer lag times while building MSMs and their effect on the kinetics have not been well studied. It should be noted that the trajectory merging and cloning processes at each resampling step make tracking the identity of WE trajectories complicated and become even more difficult as we go toward longer lag-times.

In this article, we identify an inherent bias in long lag-time MSMs built with WE due to the preferential merging of trajectories toward the stable, low-energy basins and show that it can systematically affect the transition probabilities. All weighted ensemble algorithms that incorporate trajectory merging are susceptible to this bias regardless of binning procedures, resampling algorithms, or values of parameters that affect the merging process. We propose a method to eliminate the merging bias while building MSMs using WE simulation data with lag times longer than one resampling step. We apply the method in a 1D biased random walk system and compare the time-lagged WE-MSM probability distribution against the analytical exact probabilities. The method is then applied to a complex biomolecular system of a ligand unbinding from the soluble epoxide hydrolase (sEH) protein.

## Methods

### Weighted Ensemble Methods

Weighted ensemble (WE) methods are trajectory parallelization strategies^43^ that alternate between evolving a set of trajectories forward in time and performing a statistical “resampling” process. During resampling, the aim is to shift emphasis toward undersampled regions by “cloning” certain trajectories of the ensemble. Each trajectory, *i*, in the ensemble is assigned a statistical weight (*w*_*i*_) that governs the extent to which that trajectory contributes to the computation of observables.^31^ To ensure conservation of probability, the weight of a parent trajectory (or, “walker”) is distributed evenly across the clones. Typically, WE trajectories are run with a stochastic integrator, such as the Langevin integrator, so that clones quickly diverge and explore independent paths as the simulation continues. Here, we refer to the length of these trajectory segments as τ_WE_.

To save computational expense, pairs of walkers can also be “merged” together, typically in the oversampled regions of space near local or global free energy minima. When two walkers *A* and *B* are merged, the resulting walker takes on the sum of the weights (*w*_*A*_ + *w*_*B*_), and adopts either the conformation of walker *A* (with probability *w*_*A*_/(*w*_*A*_ + *w*_*B*_)) or walker *B* (with probability *w*_*B*_/(*w*_*A*_ + *w*_*B*_)). The exact nature of this random choice is important to ensure that the expectation value of the flow of probability is zero between any two regions of space.^32^

A common, but not necessary, method of determining which walkers are cloned and merged is to divide the space into a set of bins and conduct the cloning and merging steps in a manner that makes the number of walkers as even as possible across the bins.^34^ Other algorithms for cloning and merging (also referred to as “resampling”) have also been developed, including REVO (“resampling ensembles by variation optimization”),^44^ which is used here and described below. It is worth noting that using the same argument that shows that WE is statistically exact, a corollary is that all WE resampling strategies are statistically equivalent, given that they adhere to the merging and cloning rules outlined above. The choice of algorithm thus affects only the efficiency with which a given result is obtained.

**Figure 1:**
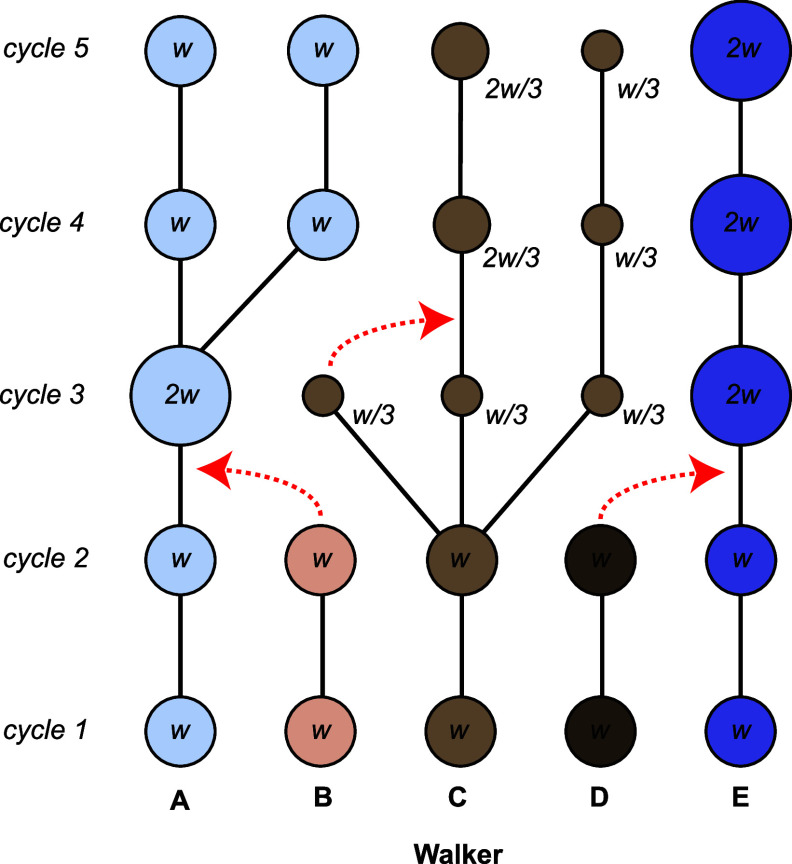
Schematic representation of resampling in the weighted ensemble algorithm. The weight of each frame is shown and also represented using the size of the circles. Merging events are shown using curved red arrows, where walkers are terminated, and their weight is added to another walker.

Analysis of WE trajectories can be complicated by the branched nature of the data set. Figure 1 shows a schematic of a “trajectory tree” that labels cloning and merging events. The tree “grows” upward from an initial ensemble. Cloning events introduce branching, and merging events result in the termination of a branch. For analyses that use time-lagged data sets, such as Markov state modeling, one must use paths in this trajectory tree to generate the time-lagged pairs. For instance, using a Markov lag time of 4τ_WE_, the trajectory tree in Figure 1 would yield five transitions: A1–A5, A1–B5, C1–C5, C1–D5, and E1–E5, where “A1” refers to the state of walker A at cycle 1. In this work, we show that this set of transitions, though constructed through intuitive means, is incomplete and systematically biased.

### REVO Resampling Algorithm

The resampling algorithm used in this work is known as REVO (resampling of ensembles by variation optimization).^44^ REVO is a bin-less resampler that aims to keep the trajectory ensemble as small as possible while sampling pathways of rare events and determining their corresponding kinetics.^39,41,44^ In REVO, merging and cloning events are coupled: for each cloning event, there is a merging event, which keeps the total number of walkers conserved. In lieu of bins, the acceptance of a combined merging/cloning operation is determined by an objective variable termed the “trajectory variation”, *V*:
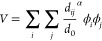
1which is evaluated both before and after the proposed events. This quantifies the variation between members of the trajectory set using a measurement of distance *d*_*ij*_. This distance metric can vary from system to system and is meant to capture important differences between walkers. The constant *d*_0_ is a characteristic distance to make the variation unitless but does not affect merging or cloning outcomes. It is calculated by running a single dynamic cycle and then estimating the average distance between all walkers. We have estimated the value of *d*_0_ to be 0.1 and 0.25, respectively, for sEH ligand unbinding simulations and 1D biased random walk simulations. The function ϕ_*i*_ determines the importance of individual walkers and is typically defined as a function of the walker weight: *ϕ*(*w_i_*) = log(*w_i_*) – log(*p*_min_/*C*), where *p*_min_ is a predefined minimum walker weight allowed in the simulation, and *C* is a constant, set here to 100 following previous works.^40,45,46^ Similarly, we have used the default values of *p*_min_ and *p*_max_ as implemented in the *REVOResampler()*. The *merge*_*d*_*ist* keyword in the *REVOResampler()* is the threshold of the distance that determines if two walkers are merged or not. In the ligand unbinding systems, we use a value 0.25 i.e., 0.25 nm of ligand RMSD. This essentially means two walkers with ligand RMSD less than 0.25 nm can be merged provided other criteria are satisfied. If the proposed cloning and merging event increases the value of *V*, then it is executed, and another coupled merging and cloning event is proposed. This process continues until *V* reaches a local maximum, at which point the resampling step is complete. One round of MD propagation, followed by a resampling step, is called a “cycle”. In general, we run a set of independent REVO simulations for a fixed number of cycles in each to gain statistical significance of the observables.

### Biased Random Walk System

To demonstrate the source of algorithmic bias, as well as our proposed solution, we first use a simple, analytically tractable system: a one-dimensional biased random walk.^44,47^ This biased random walk has 16 discrete states: *x* = 0 through *x* = 15. At each step, a walker must move from its current position (state) with a forward hopping probability of 0.25 and a backward hopping probability of 0.75. At position 0, the walker cannot go backward; therefore, the backward hopping probability is changed to a self-transition. For the final state, *x* = 15, walkers have a 0.25 forward probability of hopping back to the state *x* = 0. This is analogous to “warping” boundary conditions that have previously been used to study kinetics.^42,48,49^ Our main motivation for incorporating warping directly into the integrator, as opposed to using a boundary condition in Wepy, is to allow for consistent results to be obtained using resampling intervals longer than one cycle. We also note that the 0.25 probability of warping from state 15 to state 0 is equivalent to having a 0.25 probability of advancing to state 16 that then warps to state 0 with a full probability. The hopping probabilities can be arranged into a matrix, which is called the transition matrix (**T**). This matrix is shown graphically in Figure S1.

We have run three sets of biased random walk simulations with different merging criteria, each with 10 independent runs of 6000 cycles and 48 walkers. In the first merging criterion, referred to as “strict”, only walkers with identical *x* values can be merged. In the second merging criterion, referred to as “lenient”, walkers can be merged if |*x*_*i*_ – *x*_*j*_| ≤ 1. In the third, referred to as “straight-forward”, no merging events are allowed, resulting in 48 independent, straightforward trajectories. In each case, resampling is attempted after each biased random walk step, i.e., one step of walker movement. All simulations were carried out using the Wepy software,^50^ which is a Python implementation of the REVO resampler.

We can determine the steady state probabilities (*P(x)*) directly as the eigenvector of the transition matrix with eigenvalue 1. These show an exponentially decreasing function that is approximately linear on a logarithmic scale. This is a very simple model of a unidirectional rare event system, such as a ligand unbinding system, where a large energy barrier separates the stable (bound) state at *x* = 0 from another state at *x* = 15. The rate of this transition can be calculated as *P*(15) × 0.25, which is the steady-state flux through the boundary from *x* = 15 to *x* = 0. For this reason, apart from the accurate estimation of probabilities across all of the states, a model for kinetics can be judged by the accuracy of the final state probability.

### Ligand Unbinding from Soluble Epoxide Hydrolase (sEH)

**Figure 2:**
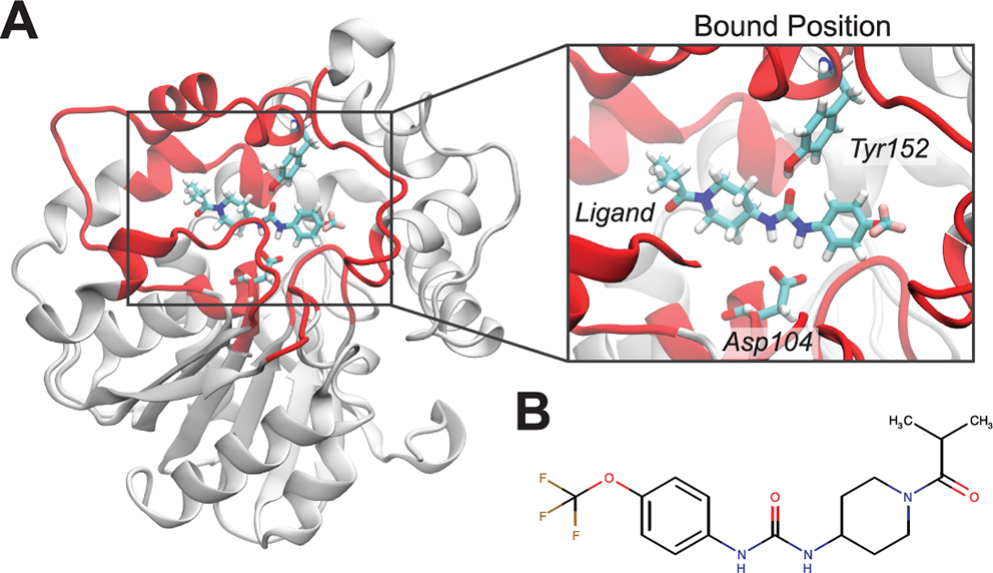
Structure of sEH and the inhibitor studied here. (A) Epoxide hydrolase domain of sEH protein (PDB ID: 4OD0). The binding region is highlighted in red, and the bound position of the ligand is shown. (B) 2D representation of the sEH inhibitor.

Soluble epoxide hydrolase (sEH) protein, a homodimeric protein present in mammalian tissues, has pharmacological relevance as a potential target in multiple diseases, including systemic inflammation, hypertension, and pain relief.^51−54^ Inhibitor design for sEH has thus gained significant attention over the years,^55^ and it has been previously shown that the ligand residence time is a key quantity for determining the efficacy of sEH inhibitors.^55,56^ Our laboratory has experience in simulating ligand unbinding from sEH^25,41^ using weighted ensemble, making it an appealing case study for benchmarking the proposed method. For this purpose, we utilize part of a WE sEH-ligand unbinding data set that we reported recently.^41^ Inhibitor 1-(1-isobutyrylpiperidin-4-yl)-3-(4-(trifluoromethoxy)phenyl)urea (hereafter referred to as the “ligand”) is our candidate of choice since it had the highest deviation in the computationally predicted mean first passage times (MFPTs) compared to its experimentally observed MFPT. Here, we focus on the MFPTs from computational models built with only ligand-binding site distance features and not with the time-lagged independent component analysis (tICA) features, as we have observed some inconsistency in our previous work with tICA.^41^ The experimentally determined half-life time ((ln 2)/*k*_off_) is 18.8 ± 0.6 min,^55^ which implies ligand MFPT in the range of 26.3 to 28.0 min. The bound pose of the ligand in the sEH protein is shown in Figure 2A, with the two primary H-bonding residues (ASP104 and TYR152) highlighted in licorice representation. The chemical structure of the ligand is shown in Figure 2B. The details of the ligand unbinding REVO simulations with a total of 17.03 μs of aggregated sampling are discussed in the Supporting Information, as well as previous work.^41^ The ligand unbinding simulations were carried out using the Wepy software.^50^

### Markov State Models

Generally, MSMs are coarse-grained dynamical models that divide a given conformation space into a finite number of states.^6−8^ In discrete-time Markov models, the evolution of the system is modeled by a transition matrix **T**(τ) whose elements *t*_*ij*_ describe the probability of the system arriving in state *j* at time *t* + τ, given that it was in state *i* at time *t*. The matrix **T** can be constructed from a counts matrix **C** that simply counts τ-lagged transitions between states by normalizing each column by its sum. A natural strategy when constructing an MSM from WE data is to use the weights associated with each walker as counts instead of 1-based counts.^41,42^ As the weight can change over the course of a τ-lagged transition, we will refer to the weight at the end of a transition as *w*2, and the weight at the beginning of a transition as *w*1. Since the weight of the trajectory is *w*2 at the time the observable is recorded, *w*2 is used for counting.

An alternative strategy is to account only for trajectory duplication. That is, if a trajectory is cloned into 4 copies by the time it reaches *t* + τ, then each copy should contribute only 1/4 to the counts matrix. This can be achieved by using the ratio of the counts: *w*2*/w*1. While the ratio-based counts are a proper depiction of the Markovian nature in a system, the *w*2-based counts can capture some non-Markovian effects as they weight transitions differently depending on their history. In practice, it has been shown that *w*2-based counts have been able to capture the non-Markovian kinetics in biologically relevant systems and provide better agreement with experimental kinetics for long timescale transitions.^41,45^ In this work, we will examine both strategies, referring to them below as either “*w*2*/w*1 counts” or “*w*2 counts”.

## Results

### Merging Bias in WE-MSMs

Even with the benefit of walker weights, using lag-times greater than one resampling step (τ_WE_) introduces significant complications in MSMs built with WE data. The problem originates with the WE walkers that are killed (squashed) within a given time interval as the outcomes of those walkers are not included in the counts matrix. If the omitted walkers were randomly distributed, this would not impart any bias to the MSM; however, this is not the case in practice. Consider a set of trajectories that all originate from an intermediate state *i*, which is high in energy. Some of these trajectories will remain in higher energy regions, while most will fall back downward toward more stable states. The trajectories that fall toward more stable states are more likely to be merged and hence be omitted from the counts matrix. Omitting the incomplete trajectories, therefore, has potential to systematically undercount transitions back toward stable states. We refer to this problem as merging bias.

**Figure 3:**
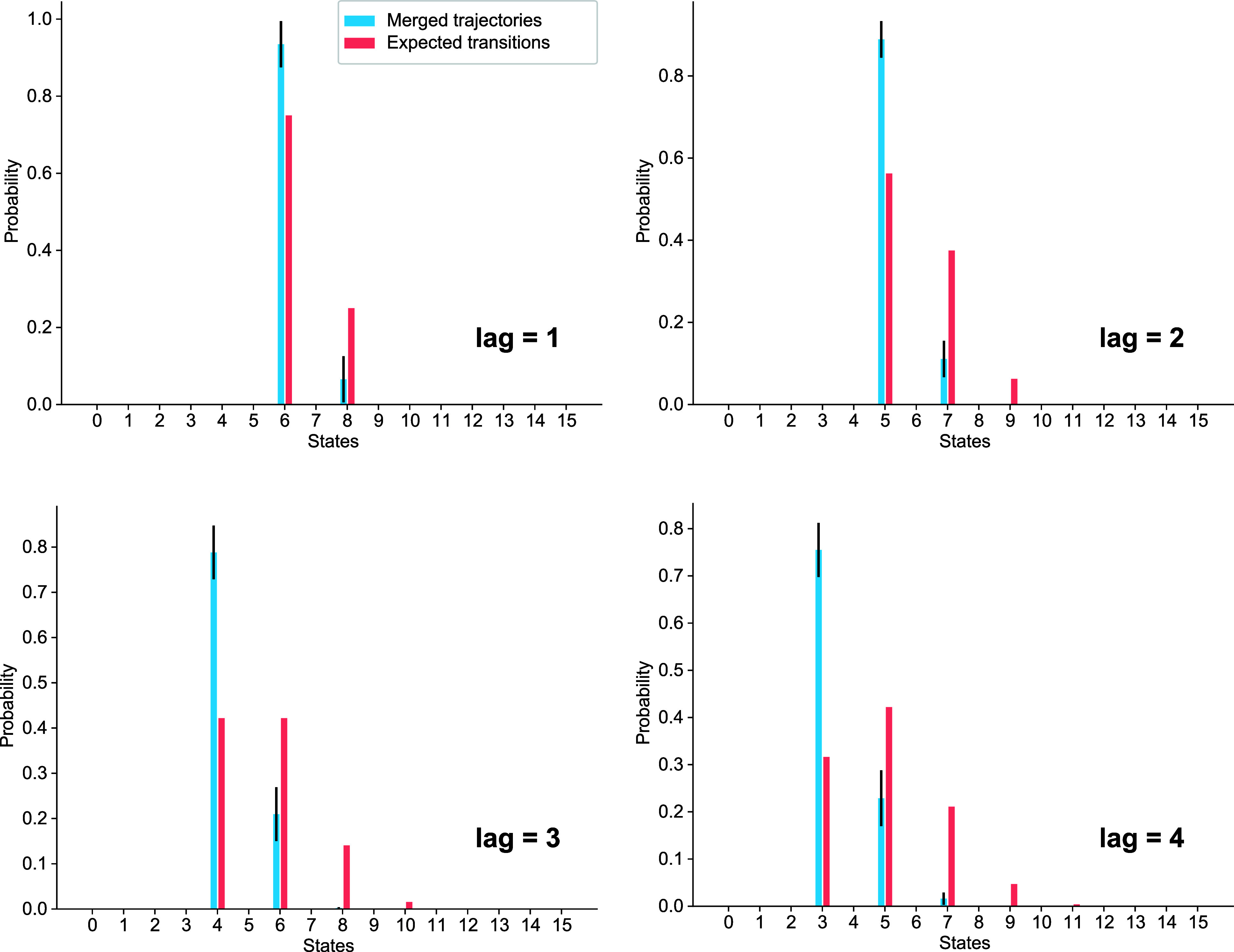
A demonstration of merging bias in a 1D biased random walk system. All panels show probability distributions of trajectories that start at *x* = 7 and have evolved for the lag time shown. In all panels, the incomplete trajectories, which were cut short due to merging events, are shown with blue bars. These can be directly compared with the analytical transition probabilities, which are shown in red.

The presence of a merging bias can be detected even in the simple 1D biased random walk (1D-BRW) system. As a reminder, the steady-state probability distribution of the 1D-BRW decays monotonically from *x* = 0 to *x* = 15. To examine the presence of merging bias, we consider the ensemble of trajectories starting at *x* = 7 that were prematurely terminated by merging in our WE simulations. Figure 3 shows the distribution of merging events in the biased random walk system collected with the lenient merging algorithm. The blue bars show where these merging events are located after the given lag time. If these were evenly distributed, this should directly match the analytical transition probabilities (red bars). For instance, after a single step (top left panel), this would be 0.25 at *x* = 8 and 0.75 at *x* = 6. However, we observe that in our WE data set, the walkers are squashed toward the left predominantly. This is in accordance with the discussion above: walkers are much more likely to be merged as they approach the more populated states. This generally becomes more prominent as the lag time increases. Furthermore, we examined the distribution of merging events in the biased random walk system after one cycle across the different starting states. Figure S2 shows that for almost all starting states there is a clear bias in the incomplete trajectory set, in that the merged trajectories are not distributed according to the expected transition probabilities. Curiously, earlier states 1 and 2 show the opposite trend in Figure S2, showing the opposite bias, with rightward trajectories being more likely to be prematurely merged. This arises due to technical reasons, where trajectories in the most probable state *x* = 0 are often unable to merge due to the maximum weight threshold, *p*_max_. We note that although the direction of merging bias might vary from state to state, any systematic exclusion of trajectories caused by premature merging can cause errors in the calculated transition matrix.

### Merging Bias Corrected MSMs (MBC-MSM)

**Figure 4:**
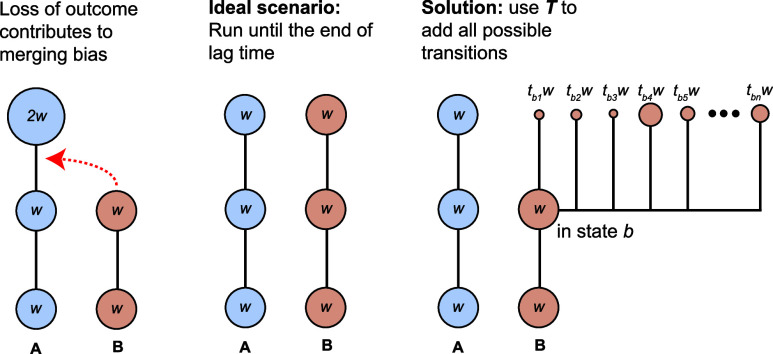
Trajectory tree segments demonstrate the merging bias correction approach. When constructing an MSM with lag time 2, the transition from trajectory B would be omitted, potentially leading to merging bias. To mimic the continuation of trajectory B, transition matrix **T** is used. Note that since the columns of **T** are normalized, the sum of all of the added trajectories is equal to *w*.

We now propose a method to eliminate the merging bias in the MSMs built with the WE data. Our aim is to account for all of the missing transitions, predict their outcomes, and add these to the corrected count matrix (Figure 4). The complete and merging bias corrected τ-lagged count matrix (**C**(τ)) can be computed as
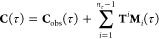
2where we denote our observed counts matrix as **C**_obs_ and the one-step transition matrix as **T**. The integer *n*_τ_ is equal to τ/τ_WE_, or the number of weighted ensemble steps in the Markov lag-time. The matrices **M**_*i*_, have elements *m*_*i(k,j)*_ that count the number of “incomplete” trajectories that have made it to state *k*, starting from state *j*, but there are still *i* timesteps left in the time interval. Note that the one-step transition matrix is exponentiated by *i*, so that when multiplied by **M**_*i*_, it propagates the data points *i* steps into the future. The summation  can be seen as a correction factor that accounts for all possible outcomes of the missing transitions. Note that the 1-step transition matrix in a WE framework is free from the merging bias or trajectory incompleteness since walkers that are merged after a single step are still accounted for. Hence, the transition matrix **T** calculated by normalizing the columns of **C**_obs_(τ_WE_) (i.e., the transition counts from one WE resampling period) is sufficient.

As mentioned in Methods section, we use the WE weights to build the C_obs_ matrix. But since we aim to determine the state-to-state transition probabilities as accurately as possible using the WE data, we make a further adjustment to the WE weights in order to remove the weight increases that result from merging. For each cycle *i*, we determine a table of walker weights *w*_*i*_ that neglects all weight increases occurring from cycle *i* onward. These are determined by iterating through the trajectory tree and dividing weights upon cloning. These weights allow us to accurately determine where the weight from cycle *i* is transported. The missing weights are then exactly compensated by the **M** matrices. Given that there are two procedures for using WE weights to calculate counts matrices (“w2-based” and “w2/w1-based”, see Methods section), it is important to note that the procedure for constructing **C**_obs_ and **M**_*i*_ should be the same.

This method of adding missing transitions recovers the MSMs from merging bias, and hence, we refer to them as merging bias corrected Markov state models (MBC-MSMs). Below, we demonstrate this approach using a set of WE simulations for both the biased random walk system and the sEH-ligand unbinding system. The code to implement MBC-MSM from a WE simulation data set in Wepy can be found in https://github.com/SamikBose/MBC_MSM.

### MBC-MSMs from REVO Biased Random Walk Simulations

**Figure 5:**
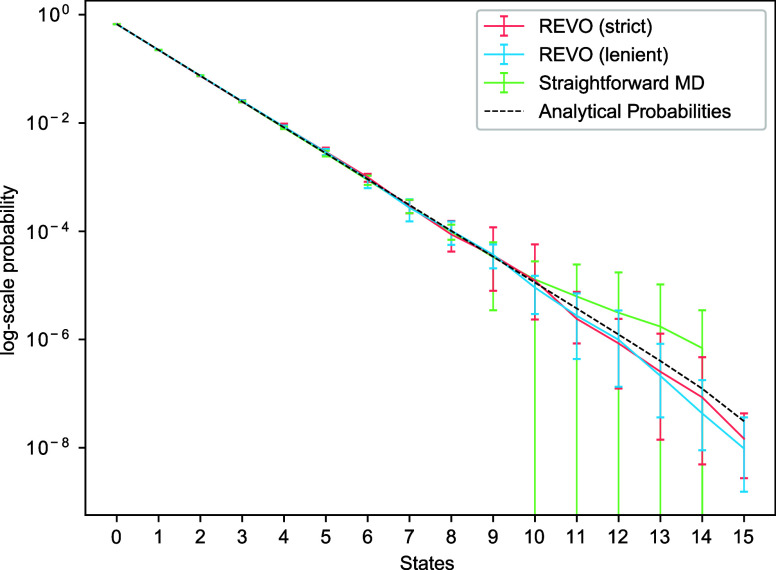
Probability distribution on a log scale across the states for a set of biased random walk simulations: REVO with a lenient resampling (in red), REVO with a strict resampling (in sky-blue), and straightforward simulation without resampling (in green). The standard errors across all the runs are shown in the error bars. The exact, analytical probabilities is plotted as the yellow line.

A set of 10 independent REVO runs is carried out, each with 6000 cycles and 48 walkers. Separate sets are run for “strict merging”, where only trajectories in the exact same state are merged, and “lenient merging”, where trajectories in the same or adjacent states can be merged. Figure 5 shows the average steady-state probabilities along with their standard errors calculated across all the WE runs in log 10 scale, compared with the “straight-forward” case, in which no resampling occurs, and the analytical solution. Generally, all methods succeed at matching the analytical probability for *x* < 10, although uncertainties grow as *x* increases. Deviations from the analytical curve increase as *x* approaches the *x* = 15 boundary. The estimated state probabilities from straightforward runs (without resampling) show the largest errors, and the final state is never populated by any walkers in any of the straightforward runs. As expected, both sets of WE simulations populate every state in the system in all of the runs. The deviation of these two from the analytical probability distribution is also significantly lower compared with the straightforward set. For lenient resampling, the root-mean-square log-errors (RMSLEs) in probabilities across all states range between 0.08 and 0.60 across 10 different runs, with mean and standard errors of 0.36 and 0.05, respectively. The strict resampling counterpart has RMSLEs ranging between 0.13 and 0.66 with mean and standard errors of 0.34 and 0.04, respectively. The performances of both resampling approaches are thus indistinguishable. We have tabulated the average state-wise probabilities of each run for all three resampling conditions in Table S2.

As discussed earlier, the final state probability is the key observable of kinetics as it is proportional to the outward flux into the “product” state. We observe that the absolute error in the final state probabilities ranges from 0.2 to 1.5 orders of magnitude for all the runs with either of the resampling strategies. The average absolute errors across all the runs are 0.5 and 0.6 orders of magnitude in lenient- and strict-resampling-based WE simulations, respectively. We now seek to discover whether this error can be reduced with the help of MSMs trained on the WE data.

**Figure 6:**
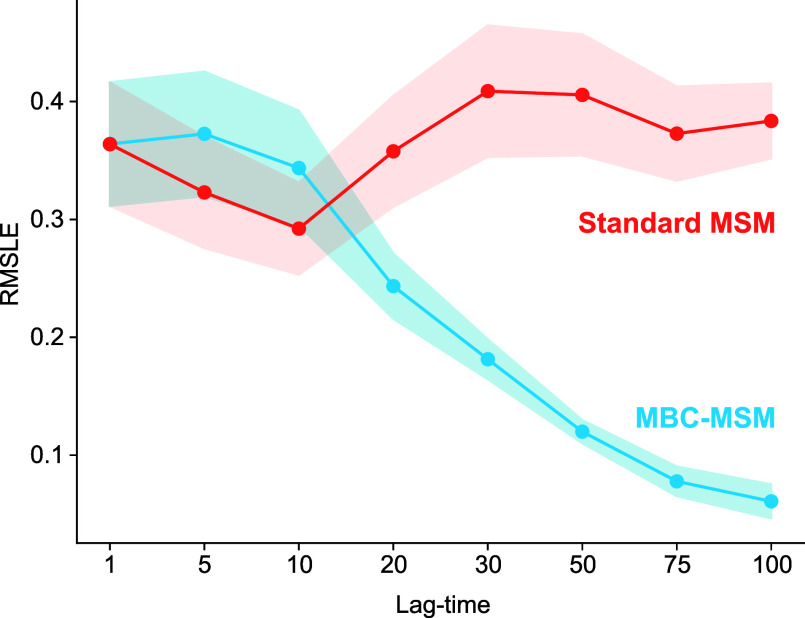
Root mean square log error in the state probabilities compared to analytical values, computed over a set of lag-times using merging bias corrected MSMs (blue) and standard MSMs (red). The shaded areas show standard errors in the predictions.

**Figure 7:**
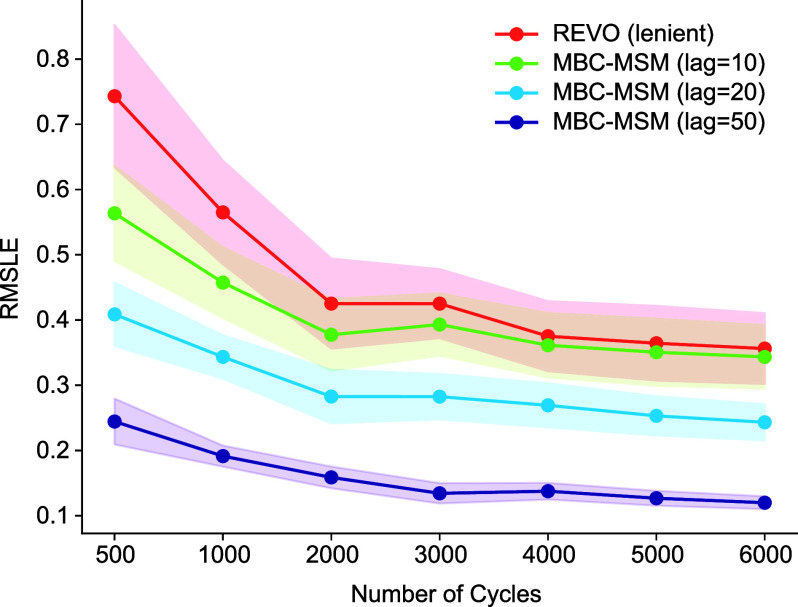
Convergence of RMSLE in the state probabilities compared to analytical values, as estimated by lenient REVO (red) and predicted by merging bias corrected MSMs built at different lag times. The shaded areas show the standard errors in the predictions.

We build both standard MSMs (using the *w*2-counts method) and MBC-MSMs, described above, using a set of lag times ranging from 1 to 100 τ_WE_, where τ_WE_ is the resampling frequency, which in this case is a single hop. For each time-lagged MSM, we compute the steady-state probability distribution and its RMSLE compared to those of the analytical solution. MSMs built with WE data from “lenient” merging are shown in Figure 6, and similar results were obtained for the strict-merging data set (Figures S3–S4). For a lag time of 1, both MBC-MSM and standard MSMs are the same, by construction, and they show a slightly improved RMSLE of 0.37, compared to the WE result of 0.39. As the lag time is increased, the RMSLEs in state probabilities of MBC-MSMs decrease sharply, while the standard MSM RMSLE is relatively constant. With lag times of 75 or 100 resampling steps, the RMSLEs are below 1/10th of an order of magnitude compared to the analytical probability distribution. The standard error across runs, which is shown by the shaded region in the figure, also reduces significantly for MBC-MSMs with the increase in lag time. This clearly demonstrates that eliminating the merging bias from MSMs built with WE data can improve the accuracy of probability distributions. Another key observation is that, similar to MSMs built on straightforward trajectories, using longer lag-times in MBC-MSM with WE data can improve the model accuracy. In examining the convergence of these results with sampling time (Figure 7), we notice that longer lag times obtain significantly lower RMSLEs with a fraction of the cycles used in a stand-alone WE simulation. For an MBC-MSM with a lag time of 50 resampling steps, ∼500 cycles obtain an RMSLE that outperforms 6000 cycles of standard WE simulation.

**Figure 8:**
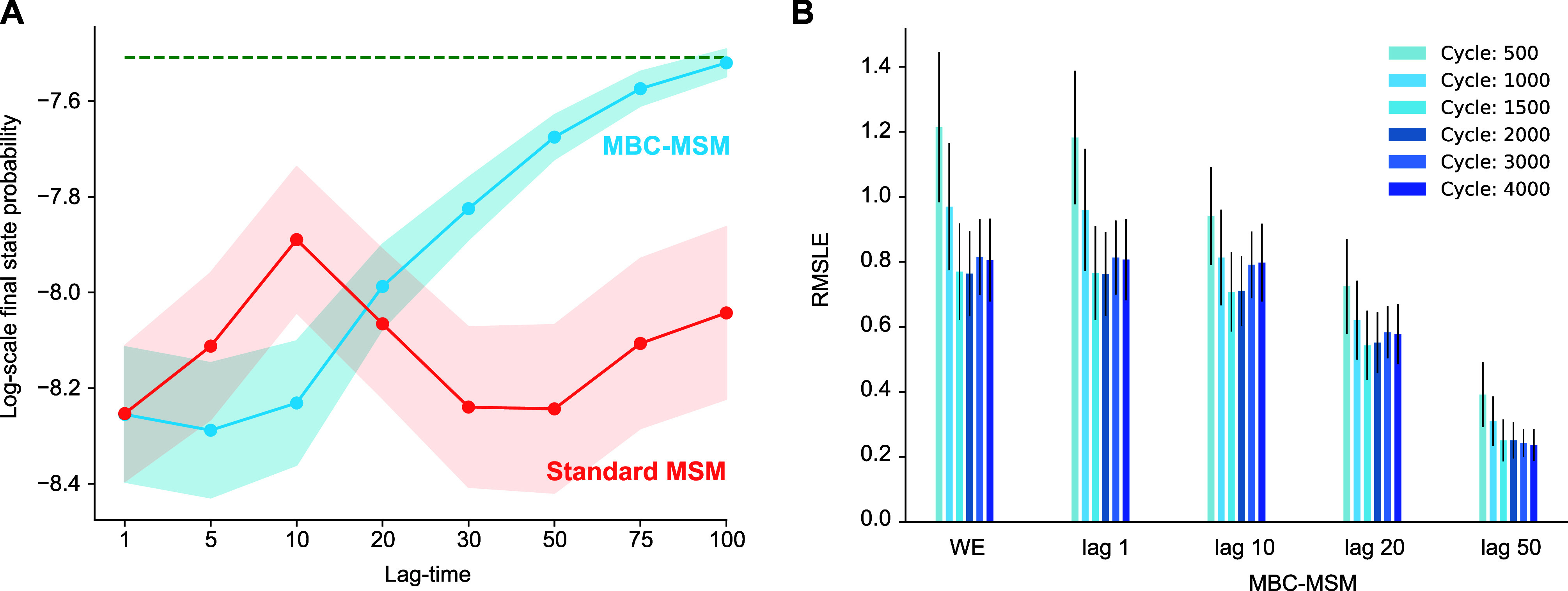
Analysis of final state probabilities in the biased random walk system. (A) Average final state probabilities for a set of lag times by MBC-MSM (blue) compared to standard-MSM (red). The dashed green line represents the exact probability of the analytical solution. (B) Convergence of the final state probabilities across a set of MBC-MSMs with different lag times. The leftmost bars show errors computed directly from WE data without a Markov state model.

The final state probability, which corresponds to the rate of crossing the boundary, is also predicted more accurately with longer time-lagged MBC-MSMs compared to its standard counterpart. As seen in Figure 8A, the predicted probability by MBC-MSM is almost identical to the analytical value (log-scale error < 0.1) at lag times of 75 or 100, with a low standard error across runs that also decreases with larger lag times. The standard MSM-predicted final state probability deviates from the analytical probability by 0.4 to 0.7 of an order of magnitude and shows no significant improvement with lag-time. Figure 8B shows the convergence of the RMSLE of the final state probability as a function of sampling time. Estimates from direct WE sampling (WE) and estimates from MBC-MSMs with different lag times both show similar trends, reaching their minimum values after approximately 1500 cycles; however, the MBC-MSMs reach lower RMSLEs. Even with 4000 cycles, stand-alone WE simulations show relatively large errors in the final state probability with considerable variability among runs.

All of the final state probabilities in Figure 8 are estimated from MBC-MSMs built with the w2-counts method. As the states used to build the Markov matrix are perfectly Markovian by definition, we would expect that w2/w1-based counts would perform similarly, if not better than the w2-based counts. The results for the overall RMSLE and the final state probabilities with w2/w1-based counts are shown in Figures S5 and S6, respectively. For both standard and MBC-MSMs, the τ = 1 results are excellent, showing very little error. As the lag time increases, significant error is introduced for the standard MSMs, but the error in the observables from the MBC-MSMs remains much smaller. Although the τ = 1 results imply that using w2/w1-based counts could be a suitable approach, we note that in more realistic systems in which transitions between coarse-grained states are not guaranteed to be Markovian, w2-based counts are vastly superior, which we show below.

### MBC-MSMs from Ligand Unbinding REVO Simulation Data

We now examine the accuracy of the time-lagged MBC-MSMs built with complex biomolecular simulation data. The details of the WE simulation data set, feature calculation, and clustering are provided in the Supporting Information. Using the procedure described above, we build the merging bias-corrected time-lagged count matrix (**C**). The **C**_obs_ and 1-step **T** matrices are built with *w*2-count approach to preserve non-Markovian characteristics. We use a variety of cluster numbers from 500 to 1200, and a set of lag-times ranging from 1 to 175 units of resampling time, i.e., 20 ps to 3.5 ns to understand the robustness of the MSMs against the model parameters. For each cluster number, clustering is repeated 10 separate times to average over uncertainties introduced by the clustering algorithm in our estimates.

**Figure 9:**
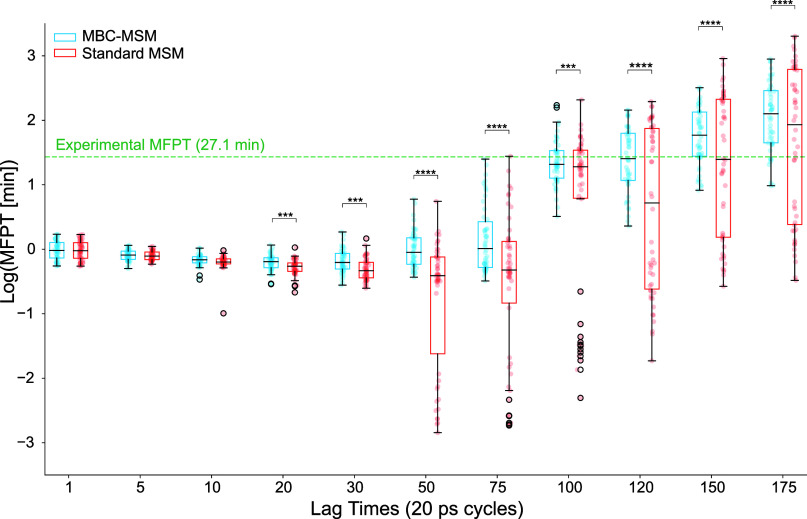
Calculated MFPTs for MBC-MSMs (blue) and standard MSM (red) as a function of lag time in the log_10_ scale. At each lag-time, several MSMs are constructed to account for variation introduced by the k-means clustering method. All results are shown as filled circles. The bar plots show the median, the interquartile range (IQR), and the whiskers extend to all points within 1.5 times the IQR. Points outside that range are shown outlined in black. The significance of differences in the accuracy of the MBC and standard MSMs are indicated by asterisks using *p* values represented as follows: * (*p* ≤ 0.05), ** (*p* ≤ 0.01), *** (*p* ≤ 0.001), and **** (*p* ≤ 0.0001). Higher numbers of asterisks denote higher significance in the accuracy difference. The *y*-axis shows the logarithm of the mean first passage time in units of minutes. An even spacing is used between differently spaced lag times in the *x*-axis for clarity in visual representation. The experimental value of 27.1 min is plotted as a green dashed line.

The mean first passage time (MFPT) of ligand unbinding is inversely proportional to the rate constant (*k*_off_) and quantifies the average time for bound-to-unbound state transitions. We use box plots to compare the MFPTs as estimated from the MBC-MSMs (in blue) and standard MSMs (in red) in Figure 9. The experimentally determined MFPT of ligand unbinding for this particular ligand has been reported earlier as 27.1 min.^55^ It is worth noting that this quantity is over 20 billion times longer than the duration of our individual WE trajectories (80 ns) and almost 100 million times longer than the combined length of all of the WE trajectories in the data set. For each lag time and MSM type, we show 30 MFPTs, calculated using 10 replicates each for cluster numbers (500, 800, and 1200). The length of the boxes shows the interquartile range (IQR, 25%–75%) of the predicted MFPTs, with the black line in the middle of each box denoting the median of all predictions at that lag-time. The whiskers extend from the edges (top and bottom) of the box to the smallest and largest data points within 1.5 times of the IQR. The length of the whiskers represents the variability in prediction, and data points outside the whiskers are the outliers. The experimentally reported MFPT is shown as a dashed line in the figure.

We observe that at shorter lag times (<50 cycles), MBC-MSMs and standard MSMs have a similar trend in MFPTs with comparable medians and IQRs. With the lag time of τ_WE_ = 20 ps, the error in predicted median MFPT by either of the methods is over an order of magnitude compared to the experimental value. MBC-MSMs at longer lag times have better accuracy in MFPT prediction compared to shorter lag times and show better accuracy than long lag-time standard MSMs. We examined the *p*-values from two-sample *t*-tests of independence to understand the statistical (in)dependence of the predicted MFPTs from standard and MBC-MSMs at each lag time. The resulting *p*-values (Table S4) along with lower mean absolute error indicate that the MFPTs from MBC-MSMs (≥ 20τ_WE_) are significantly more accurate than those from standard MSMs. It should be noted that all MSMs built for this study have the same underlying WE simulation data, irrespective of lag time and bias correction. Hence, incorporating the missing transitions in the transition probability matrices improves the accuracy of MBC-MSMs with longer lag times. The asterisk (s) along the boxes in Figure 9 denote the level of significance in the accuracy difference. The IQR of MBC-MSM MFPTs is around an order of magnitude at higher lag times (≥ 50τ_WE_), while the corresponding standard MSMs have IQRs > 4 orders of magnitude. The fact that we use a wide range of cluster numbers (from 500 to 1200) and still observe predicted MFPTs within an order of magnitude of each other validates the robustness of the MBC-MSM compared to the standard approach. Moreover, Figure S7 shows the convergence of MFPTs estimated by different approaches, such as directly from WE flux, from standard, and MBC-MSMs at different lag times. The direct WE MFPT estimate is consistent between 0.5 and 5 min, approaching a final value of 3.5 min at 3500 cycles. For a lag time of 1, the standard and MBC-MSMs are equivalent and follow a similar pattern to the direct WE estimate but are consistently faster by a factor of 3. However, at longer lag times τ_WE_ = 100, MBC-MSM and standard MSMs show significantly different convergence trends, with MBC-MSM reaching values that are more consistent with experimental results and achieving a lower standard error between clustering runs compared to standard MSMs. These results demonstrate that MBC-MSMs can produce more accurate and robust rate predictions with lower variability compared to standard MSMs, validating its use for kinetic and pathway modeling of rare biomolecular processes. The results in Figure 9 are built using w2-based counts. In Figure S8, we show the w2/w1-based counts added for comparison. Regardless of lag time, these are 6 to 8 orders of magnitude faster than rates obtained using the w2-based counts. Interestingly, the MBC-MSM models with w2/w1 counts still show smaller variation with respect to their uncorrected counterparts.

**Figure 10:**
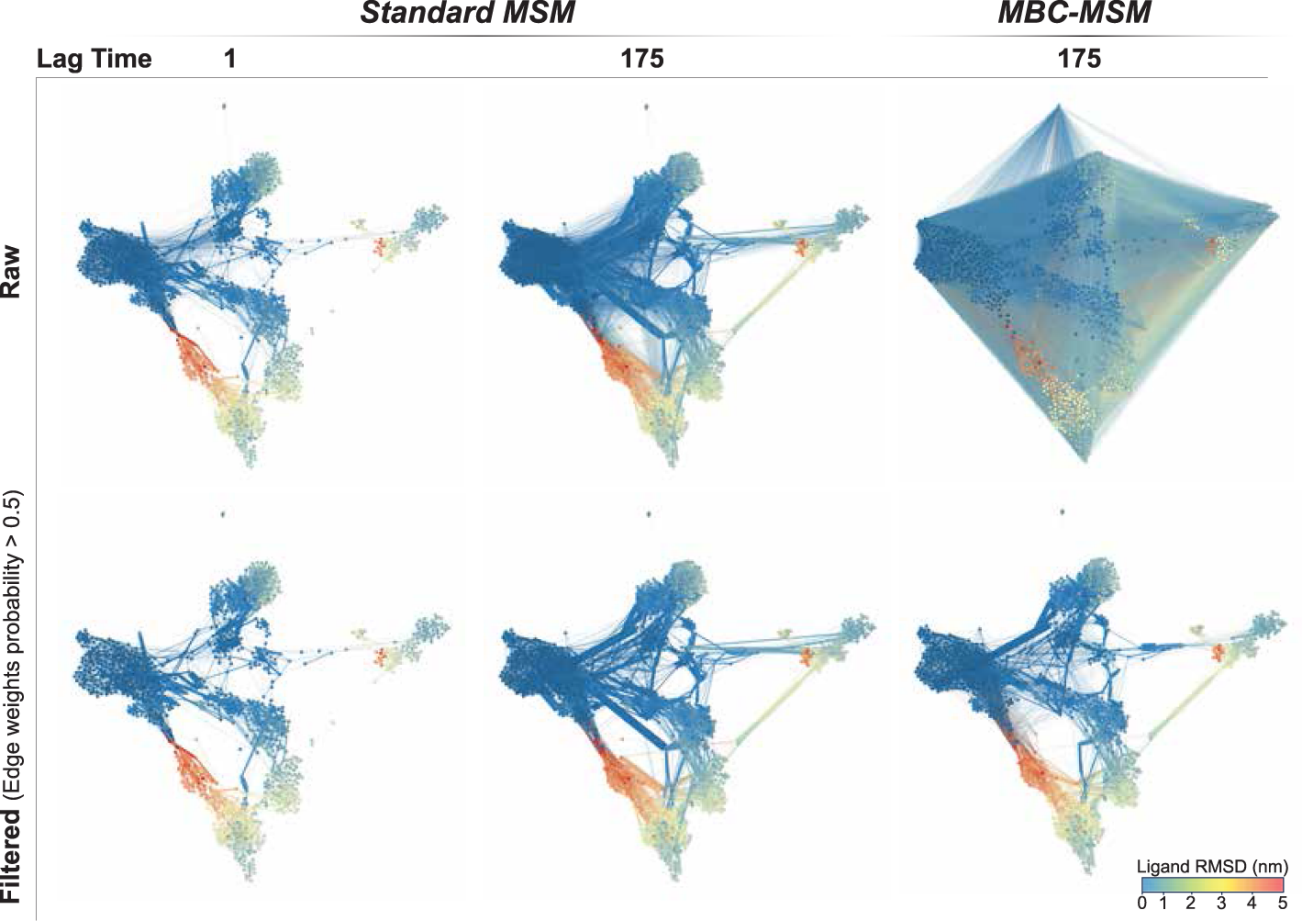
CSNs of ligand unbinding from sEH obtained by different MSM methods. The left column shows CSNs obtained with a lag time of 1, the middle column shows a standard MSM with a lag time of 175, and the right column is an MBC-MSM with a lag time of 175. The top row shows edges for every off-diagonal element in the corresponding transition matrix, and the bottom row only shows edges for transition probability values of >0.005. In all cases, networks are colored based on the ligand RMSD, with dark blue and red representing the bound and unbound states, respectively. The same node positions are used for each network plot.

To visualize differences in the transition matrices, we use conformational space networks (CSNs). These are visual representations of Markov models where each node represents a state and each edge represents an off-diagonal element of **T**. One can use various color schemes to show how average properties (such as the committor or the native RMSD) change in different regions of the network. In Figure 10, we visualize the MBC-MSMs and standard MSMs built with the ligand unbinding WE simulation data, divided into 1200 clusters. As expected, we see an increasing number of connections as the lag time increases. This is because states that are not directly connected with a lag time of 1 can become connected at higher lag-times. For MBC-MSMs, the lag-time 175 matrix becomes extremely dense: the 1200 by 1200 matrix has 636,059 nonzero elements, or roughly 44% of the total. In comparison, the standard lag-time 175 MSM has 30,408 nonzero elements, or 2% of the total. However, most of the MBC-MSM connections are of low probability. The bottom row shows the filtered networks, where only edges corresponding to transition probabilities greater than 0.005 are shown. After filtering, the number of edges is similar in both networks: 10,518 for MBC-MSM and 10,863 for the standard MSM. Interestingly, although the qualitative features of the lag time of 175 MSMs look similar, there are noticeable differences in the edge weights of the two CSNs that can affect the calculation of MFPTs. This implies that even for mechanisms and pathways, one may observe some differences with a time-lagged MBC-MSM analysis.

## Discussion

Modeling long timescale events in complex biomolecular systems with statistical significance and robustness has gained significant attention recently.^57,58^ In this regard, Markov state modeling has been instrumental in analyzing long timescale MD data and deciphering slower kinetic modes and their mechanisms. Using adaptive sampling methods,^59^ MSMs have been used to efficiently generate long timescale processes as well. Similar to WE-based enhanced sampling methods, adaptive sampling by MSMs requires examining the ensemble of trajectories after certain time intervals to decide upon the future of the trajectories. The ensemble is iteratively propagated with structures from states identified by on-the-fly MSMs that are profitable for continued investigation. In WE methods, the “resampling” is typically done at much shorter time intervals compared to adaptive sampling as the computational cost of calculating trajectory variation is significantly lower than building an MSM at each time interval. One prominent advantage of WE is the weights associated with the trajectories that are carried along the trace from the beginning of the simulation. These weights can be directly utilized to count the transitions and contain some information about the history of the trajectories. The recent developments in non-Markovian approaches have shown the benefits of incorporating history into Markov models, and the use of WE weights can be seen as another example along these lines.^60,61^ Based on our results here, we suggest using “w2-counts” to build MSMs when using WE simulation data to help account for non-Markovian effects.

Building time-lagged WE-MSMs requires careful consideration of the resampling events along each trajectory within that lag-time. The sliding_windows module of Wepy software can be used to build time-lagged data points following the resampling in the WE trajectory ensemble. However, simply using time-lagged data from sliding_windows, without eliminating merging bias, one would incur significant errors, as seen in the prediction of final state probabilities in biased random walk and MFPTs of the ligand unbinding in sEH. The proposed MBC-MSM method eliminates merging bias and predicts transition rates in both systems with higher accuracy and robustness compared to the existing implementations of MSM with WE data. The treatment of trajectory merging in the MBC-MSM method leads to additional state-to-state transitions that are otherwise systematically undercounted in the standard WE-MSM framework. The additional transitions split the counts into multiple lower-weight transitions in the MBC-MSM. Hence, there is an increase in the number of connections between the states in the MSM with longer lag times. Collectively, these additional transitions lead to more accurate estimates of kinetic observables in long time-lagged MBC-MSM compared to standard MSMs.

The accuracy and robustness of MBC-MSM are more remarkable at longer lag times (>50 resampling steps) compared to shorter ones. This is true for both the biased random walk and the ligand unbinding from sEH systems. We believe this is due to the fact that there are a greater number of merging events possible as we increase the lag time and that the merging bias grows nonlinearly with lag-time. For a 1D biased random walk system, longer time-lagged MBC-MSM could predict the kinetically relevant and most susceptible final state probability within 0.05 of an order of magnitude of the analytical, exact probability. With a lag time of one resampling step, the absolute error is ∼0.7 of an order of magnitude from the exact probability. For the ligand unbinding example, the MSMs built with existing implementations have a median at ∼1.2 orders of magnitude from the experimental estimate, which is also consistent with previous work.^25,41^ The MBC-MSMs with lag times >100 resampling steps have medians ranging from 0.2 to 0.5 orders of magnitude from the experimental value. Based on these results, we suggest that similar to MSMs with straightforward MD simulations, one should consider utilizing higher lag times for MSMs built with WE simulation data.

However, it is also possible to choose a lag time that is too high. Most obviously, the lag time (τ) cannot be larger than the duration of the trajectories used in each run of the underlying WE data set (*N*_c_τ_WE_, where *N*_c_ is the number of cycles in the WE run). Even for values of τ that are less than, but comparable to *N*_c_τ_WE_, the number of truly independent data points in the counts matrix will be very small. The largest τ examined here for the ligand unbinding data set was between 0.05 and 0.10 of the value of *N*_c_τ_WE_ for the individual runs. We caution against using τ values larger than this without further testing. Another thing to consider is the desired temporal resolution of the model. By construction, models built with longer lag times lack the ability to describe dynamics on timescales shorter than τ. While this would not affect the accuracy of calculation of long timescale processes, it could affect the resolution of transition pathways determined by the model. Finally, the procedure described here involves constructing separate matrices **M**_*i*_ for each step 0 < *i* <τ/τ_WE_. Although improvements to our current algorithm are possible, this can become computationally costly as τ/τ_WE_ increases beyond 200.

During the modeling of ligand unbinding kinetics in sEH, we observe the median of a standard MSM with τ = τ_WE_ at 0.96 min, while longer time-lagged MBC-MSMs have a median of ∼50 min. Interestingly, the counts-filtered networks of MBC-MSM are qualitatively quite similar to the standard MSM with τ = τ_WE_, although we do see some small differences. It is thus possible that the respective transition states may have different attributes, as well. This requires careful characterization, which is beyond the scope of this work. However, the improved agreement with both analytical probabilities (for the 1D biased random walk) and experimental quantities (for the ligand unbinding system) supports the idea that transition states computed with MBC-MSMs could only improve in accuracy.

## Supporting Information Available

Details of ligand unbinding simulations, state-wise probabilities for 1D random walk, merging bias chart for difference 1D random walk starting positions, analysis of root-mean-square log error (RMSLE) and final state probability for strict merging criterion, analysis of RMSLE and final state probability for w2/w1 counts, *p*-values for MFPT comparisons in ligand unbinding system, convergence analysis of unbinding MFPT, and MFPTs with w2/w1 counts for ligand unbinding system (PDF)
