# Supplementary material for: Markov State Models with Weighted Ensemble Simulation: How to Eliminate the Trajectory Merging Bias
Source: J Chem Theory Comput. 2025 Feb 11;21(4):1805–16. doi: 10.1021/acs.jctc.4c01141 (PMC11866749; doi:10.1021/acs.jctc.4c01141)
Supplement: Supplementary file 1 — ct4c01141_si_001.pdf [file ct4c01141_si_001.pdf]

Supplementary Information: Markov State Models with Weighted Ensemble Simulation: How to Eliminate the Trajectory Merging Bias

Samik Bose,[†] Ceren Kilinc,[†] and Alex Dickson[*,†,‡]

†*Department of Biochemistry and Molecular Biology, Michigan State University, East Lansing, Michigan 48824, USA*

‡*Department of Computational Mathematics,Science and Engineering, Michigan State University,East Lansing, Michigan 48824, USA*

E-mail: alexrd@msu.edu

Soluble epoxide hydrolase ligand unbinding simulations

The details of the sEH ligand unbinding system have been reported previously by our group.[1] Here we provide a brief overview of these details, focusing on aspects that are relevant to the current work. The simulations use the structure of sEH from (PDB ID: 4od0),[2] and generate an initial ligand pose by mapping our ligand (1-(1-isobutyrylpiperidin-4-yl)-3-(4-(trifluoromethoxy)phenyl)urea) to the PDB ligand (1-trifluoromethoxyphenyl-3-(1-propionylpiperidin-4-yl)-urea), also known as TPPU. The ligand here is referred to as "Lig 4" in our previous work.[1] Only the epoxide hydrolase domain (231-547) of sEH was simulated. The protein and ligand system was prepared using CHARMM-GUI[3] and solvated with TIP3 waters, with 150 mM KCl, including extra potassium ions for charge neutralization. The length of the simulation box was set to 78 Å in each dimension. The simulations were run with the CHARMM36 force field[4] and the ligand was parameterized using CGenFF.[5] Simulations were run using the OpenMM dynamics engine,[6] using a Monte Carlo barostat, a Langevin Integrator, a force switching algorithm for nonbonded forces (10 Å - 12 Å), and a 2 fs integration step size.

The Resampling of Ensembles by Variation Optimization (REVO) resampling method[7] was used as implemented in Wepy.[8] The details of the parameters are provided in the Methods section of the article. The resampling algorithm was guided by the `UnbindingDistance` method from wepy, which measures the distance between two trajectories in the ensemble as the RMSD between ligand atoms after aligning the receptor atoms to a common reference. The set of receptor atoms are defined as those within a cutoff of 5.0 Å from the ligand in the equilibrated bound pose. The simulations were run in the "unbinding ensemble", which is a non-equilibrium ensemble where trajectories that enter the unbound state are immediately transported back to the bound state. This is achieved using the `UnbindingBC` boundary conditions method from wepy, where the unbound state is reached when the minimum protein-ligand distance exceeds 10 Å.

Five REVO simulations were conducted for this ligand, with 48 walkers in each (Table S1).

The number of resampling cycles in a run varied from 2799 to 4000. In each cycle, 10000 MD steps of dynamics (20 ps) were run for each of the 48 walkers. Given the large size of the sEH-ligand systems, the length of a simulation is often restricted by the computational resources. The wall-time of the ligand 4 unbinding simulations in our previous work is around 15 days in 8 GPUs (Nvidia RTX 2080s) for 1 run with 48 walkers and 3500 cycles i.e., 3.36 µs. We conducted 5 such runs with approximately similar length in order to determine averages and uncertainties. The results presented multiple independent observations of ligand unbinding with a transition flux that did not change by more than a factor of five over 3000 simulation cycles. We chose to stop collecting data at 3500 cycles for this reason, although it is possible that new pathways might be found with additional replicates or longer simulations. For the randomwalk simulations we have used a much longer number of cycles (6000), which is much less computationally demanding. We have used 48 walkers for both the simulations in this work. A big motivating factor is that the number of walkers should be divisible by the number of GPUs (i.e., 8 in our case), so that each GPU is loaded with exactly same number of walkers. Increasing the number of walkers adds more variation among the trajectories but entails more computational cost per cycle. We have previously found 48 walkers to be a good balance between these considerations [1,7], we have used the same here.

Table S1: Ligand unbinding REVO Simulation details. The number of unbinding events is the total number of trajectories that met the 5 Å ligand-receptor clearance criterion during the REVO simulation.

Num. walkers	Run index	Number of cycles	Aggregated sampling (μs)	Num. of unbinding events	Cycle index of unbinding event(s)
48	0	4000	3.84	5	420, 430, 707, 914, 3258
48	1	4000	3.84	2	2547, 3222
48	2	3414	3.28	1	3404
48	3	2799	2.69	0	-
48	4	3518	3.38	0	-

A set of 336 features consisting of interatomic distances between backbone atoms in the sEH binding site and ligand atoms are calculated for all frames in each trajectory and used as feature dataset to build MSMs. These features were clustered into 500, 600, 800, 1000 and 1200 number of states using the k-Means algorithm in the python scikit-learn package.[9] State labels were used along with time-lagged transitions to build counts matrices, as described in the main article.

Table S2: State-wise probabilities for the 1D biased random walk. The analytical probabilities were calculated using the first eigenvector of the transition matrix and can be considered exact. Both "lenient" and "strict" REVO probabilities were calculated using averages across 10 independent runs. The straightforward MD average probabilities were also calculated using averages across 10 independent runs. The state $x = 15$ was not visited by any of the straightforward runs.

State index	Analytical Probability	Lenient REVO average probability	Strict REVO average probability	Straighforward MD average probability
0	6.666e-01	6.670e-01	6.656e-01	6.667e-01
1	2.222e-01	2.218e-01	2.221e-01	2.223e-01
2	7.407e-02	7.365e-02	7.439e-02	7.424e-02
3	2.469e-02	2.467e-02	2.512e-02	2.459e-02
4	8.230e-03	8.448e-03	8.484e-03	8.096e-03
5	2.743e-03	2.922e-03	2.878e-03	2.705e-03
6	9.145e-04	9.939e-04	9.201e-04	8.823e-04
7	3.048e-04	2.855e-04	2.705e-04	2.868e-04
8	1.016e-04	8.712e-05	1.026e-04	9.792e-05
9	3.385e-05	3.587e-05	3.694e-05	3.333e-05
10	1.127e-05	1.229e-05	9.117e-06	1.285e-05
11	3.748e-06	2.413e-06	2.753e-06	6.250e-06
12	1.239e-06	8.494e-07	9.915e-07	3.125e-06
13	4.027e-07	2.547e-07	2.131e-07	1.736e-06
14	1.239e-07	8.565e-08	4.328e-08	6.944e-07
15	3.097e-08	1.472e-08	9.703e-09	0.000e+00

Table S3: Number of total merging events in each state that is used to compute the distribution of merging events after a lag-time 1 WE step in 1-D biased random walk system in Fig S2.

State index	Number of merging events
0	20244
1	9530
2	4053
3	5939
4	12741
5	16780
6	22888
7	26639
8	19175
9	7576
10	2579
11	834
12	343
13	135
14	42
15	0

Table S4: Illustration of estimated the p-values at specific lag-times with MBC and standard MSMs using two sample t-tests of independence. Also, the MSM with lower MAE in MFPT prediction and significance of accuracy difference is provided here. At each lag time there are (both standard and MBC) MSMs with 5 different numbers of clusters (500, 600, 800, 1000 and 1200) and for each cluster number there are 10 independent clusterings with random seeds. Hence for each lag time MBC or stanadard MSM samples for t-test are built with absolute error in MFPT from 50 MSMs. Here, the MAE (i.e., the measure of accuracy) is defined with respect to the experimental result using the average deviation calculated across the set of 50 MSMs.

Lag time (in τ_{WE})	p-value based on absolute error abs(log(COMP) - log(EXPT))	Lower MAE (MBC or standard MSM)	Signficant difference in accuracy?
1	0.9065	MBC-MSM	No
5	0.7073	MBC-MSM	No
10	0.1346	MBC-MSM	No
20	2.9e-03	MBC-MSM	Yes
30	7e-04	MBC-MSM	Yes
50	8.3e-07	MBC-MSM	Yes
75	2e-04	MBC-MSM	Yes
100	5e-04	MBC-MSM	Yes
120	7.8e-09	MBC-MSM	Yes
150	1.2e-08	MBC-MSM	Yes
175	2.8e-05	MBC-MSM	Yes

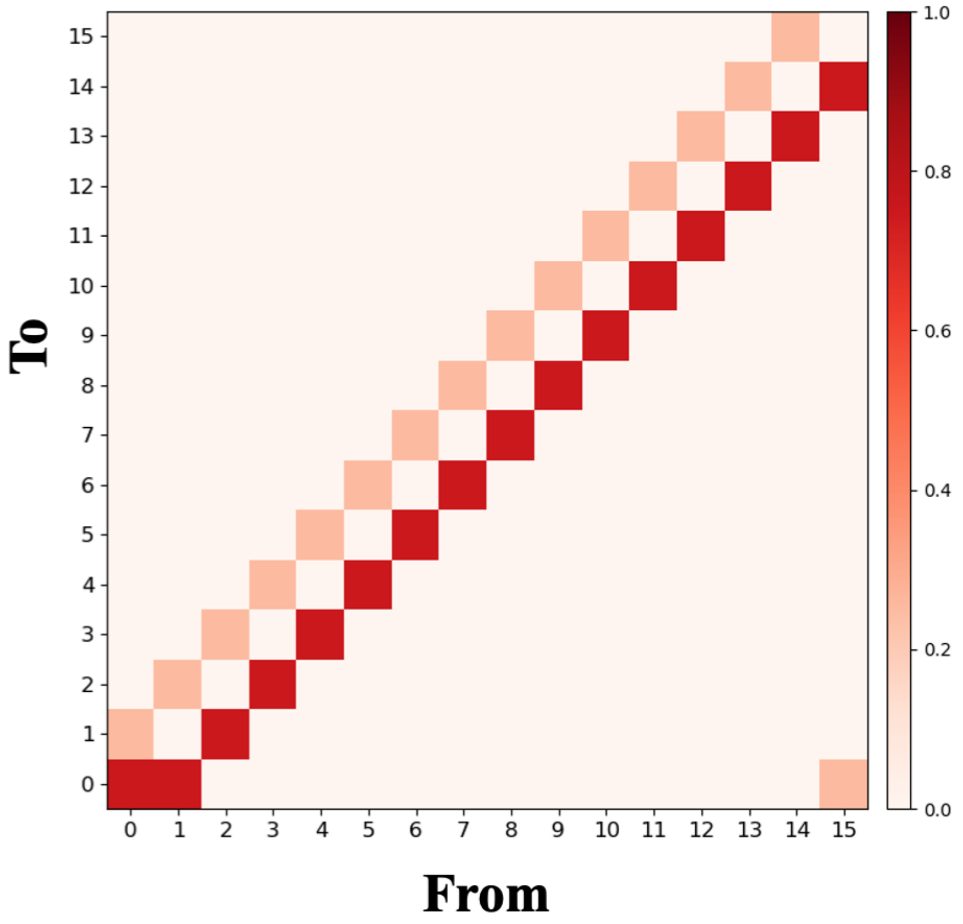

Figure S1: The 1-D biased random walk 1-step transition matrix calculated from the analytical transition probabilities.

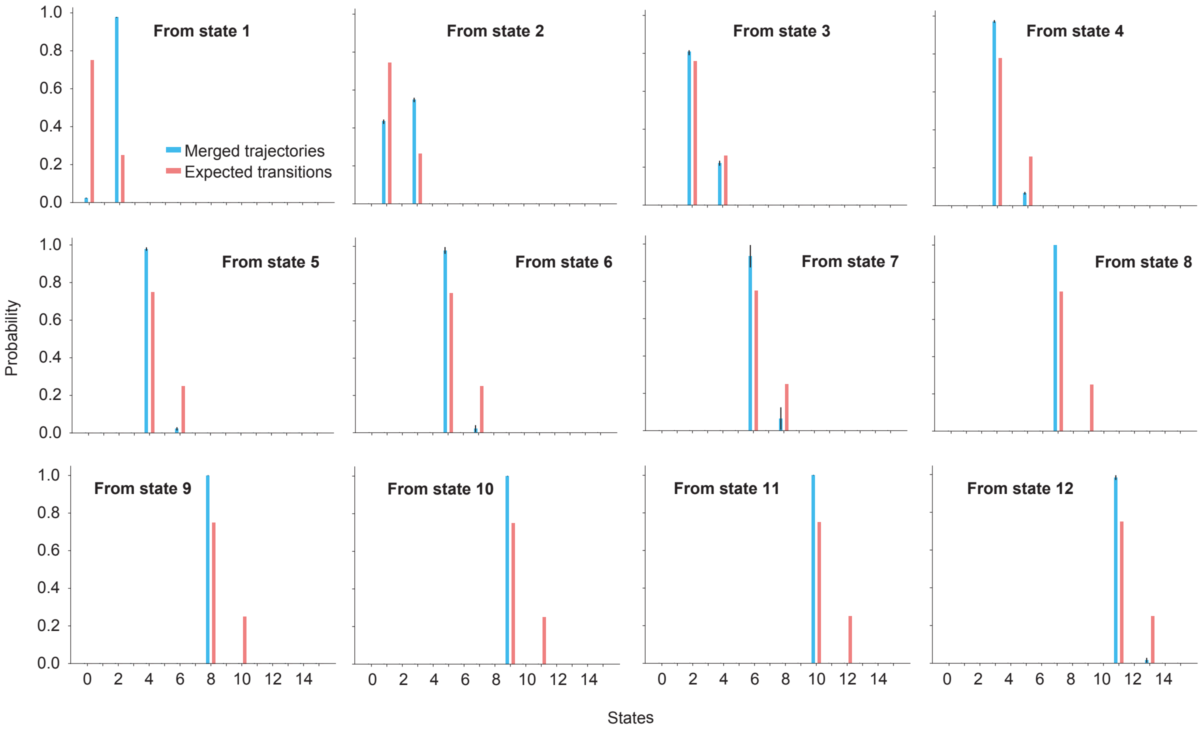

Figure S2: Merging bias in the 1D biased random walk system for different starting positions. All panels show probability distributions of trajectories that start at a specific state and have evolved from that state after a lag-time 1 step. The distribution of incomplete trajectories due to merging are shown with blue bars. The analytical transition probabilities (expected transitions) are shown in red.

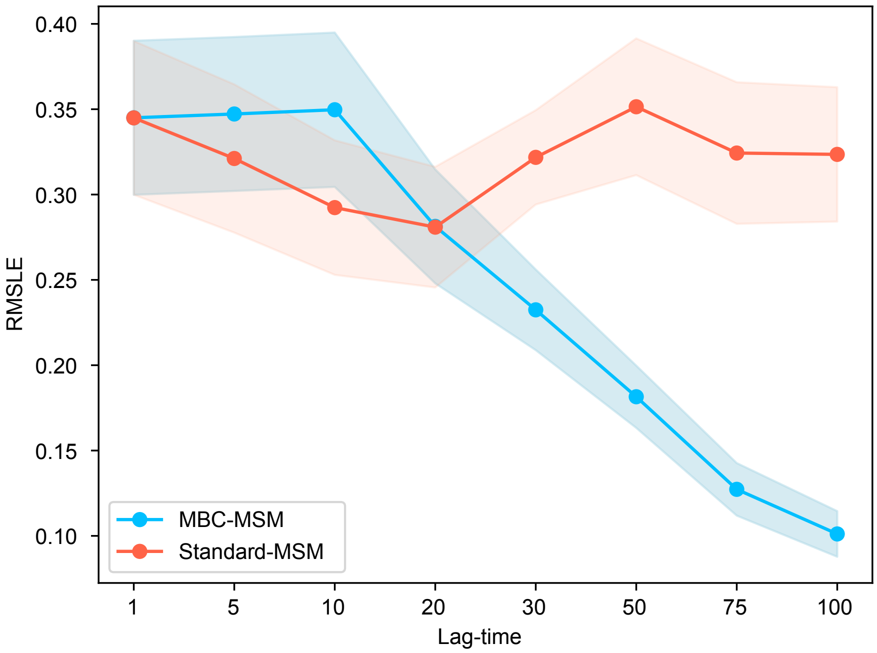

Figure S3: Root mean square log error in the state probability compared to analytical values, over a set of lag-time by merging bias corrected MSMs (blue) and standard MSMs (red) built with strict REVO resampling criteria. The shaded areas show the standard errors in the predictions.

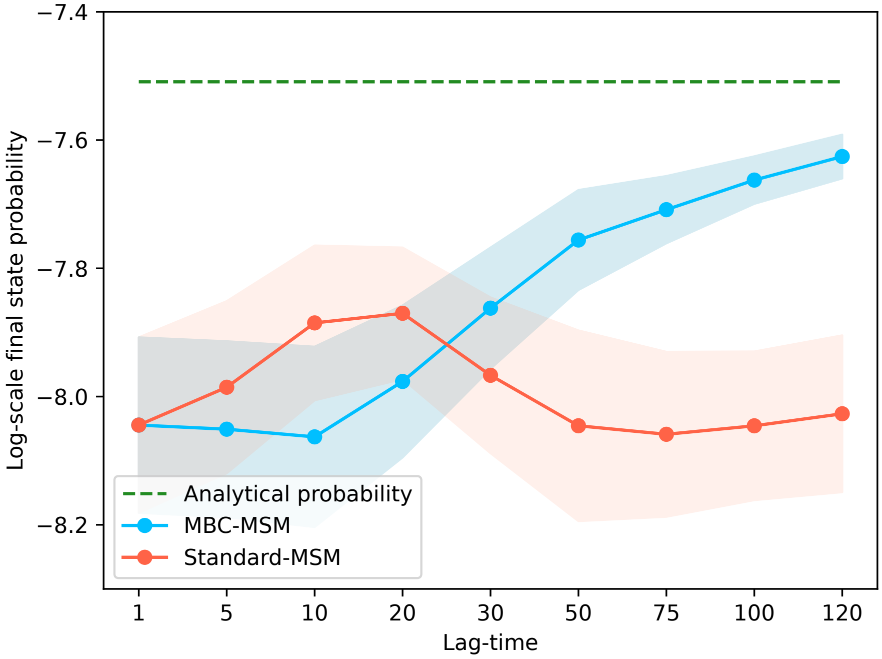

Figure S4: Probability of the final state in log-scale for a set of lag-times by MBC-MSM (blue) compared to standard-MSM (red) with strict REVO. The dashed green lines represent the exact probability from the analytical solution.

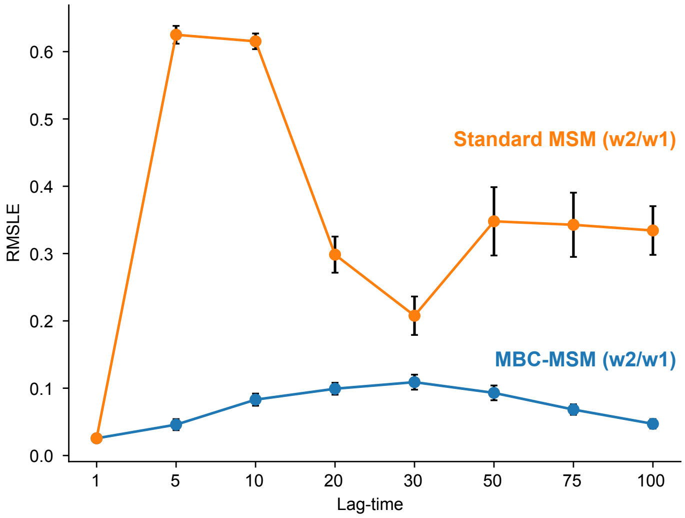

Figure S5: Average root mean squared log error for all states in the 1D biased random walk. Merge-bias corrected MSM results are shown in blue and the uncorrected MSM results are shown in orange. Error bars show standard error of the mean computed across 10 independent replicates.

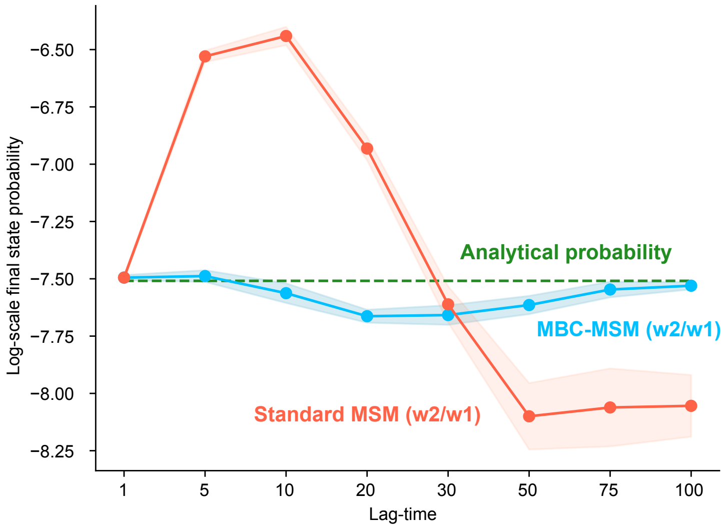

Figure S6: Average log-scale final state probability for Markov state models calculated with standard (orange) and merge-bias correction (blue) as a function of lag-time. The analytical value of the final state probability is shown as a dashed green line. Shaded areas show the standard error of the mean computed across 10 independent replicates.

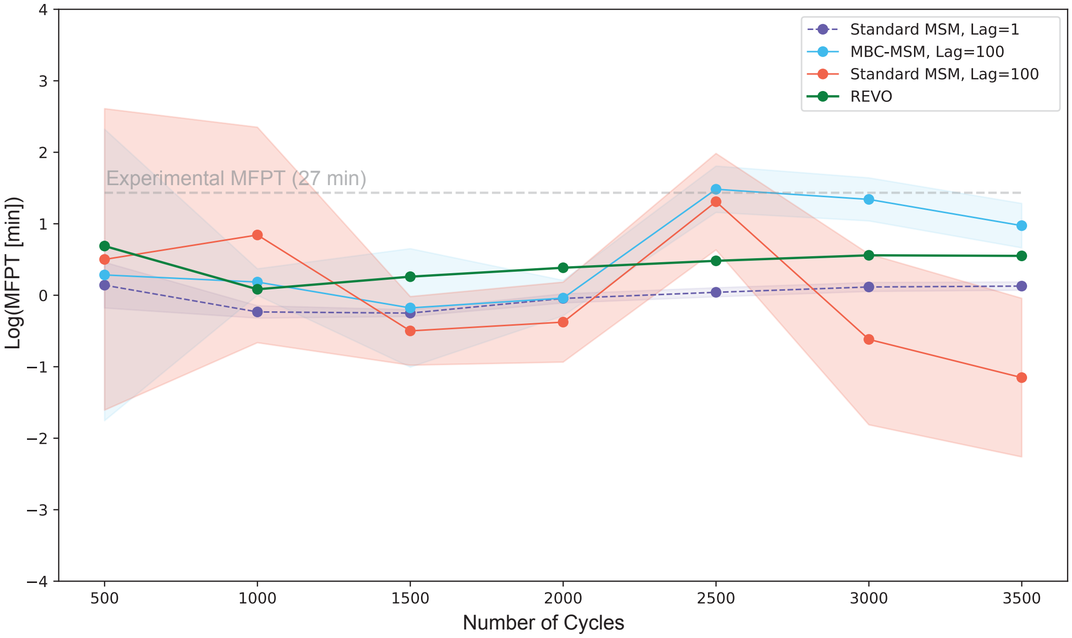

Figure S7: Estimates of the unbinding MFPT as a function of simulation time (number of WE cycles). Each symbol integrates sampling of 5 independent replicates simulated up to the specified time point. The shaded areas indicate standard errors calculated using multiple independent clusterings. Each MSM is built using 1200 clusters. The experimental value of 27.1 minutes is shown as a dashed gray line.

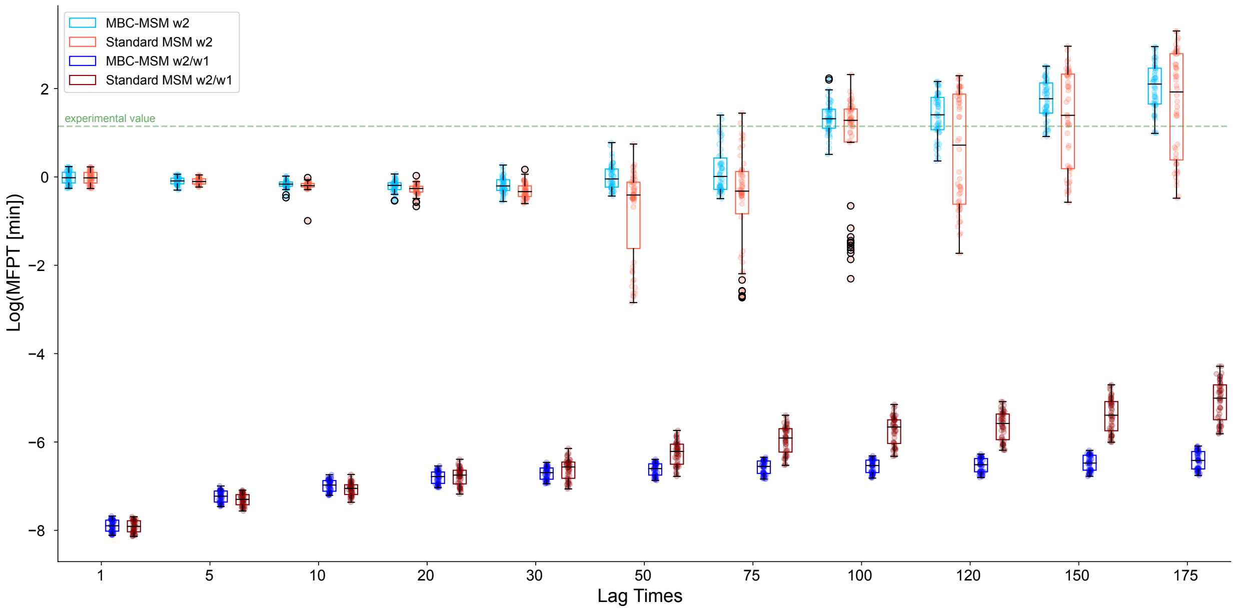

Figure S8: Mean first passage times for sEH ligand unbinding (in $\log_1 0$ scale, minutes) with w2/w1 counts results shown for comparison. Similar to the w2-count results, these include 10 replicates for Markov models built using cluster numbers 500, 800 and 1200.

References

(1) Bose, S.; Lotz, S. D.; Deb, I.; Shuck, M.; Lee, K. S. S.; Dickson, A. How Robust Is the Ligand Binding Transition State? *Journal of the American Chemical Society* **2023**, *145*, 25318–25331.

(2) Lee, K. S. S. et al. Optimized inhibitors of soluble epoxide hydrolase improve in vitro target residence time and in vivo efficacy. *Journal of Medicinal Chemistry* **2014**, *57*, 7016–7030.

(3) Jo, S.; Kim, T.; Iyer, V. G.; Im, W. CHARMM-GUI: A web-based graphical user interface for CHARMM. *Journal of Computational Chemistry* **2008**, *29*, 1859–1865.

(4) Huang, J.; Mackerell, A. D. CHARMM36 all-atom additive protein force field: Validation based on comparison to NMR data. *J. Comp. Chem.* **2013**, *34*, 2135–2145.

(5) Vanommeslaeghe, K.; Hatcher, E.; Acharya, C.; Kundu, S.; Zhong, S.; Shim, J.; Darian, E.; Guvench, O.; Lopes, P.; Vorobyov, I.; Jr., A. M. CHARMM General Force Field (CGenFF): A force field for drug-like molecules compatible with the CHARMM all-atom additive biological force fields. *Journal of Computational Chemistry* **2010**, *31*, 671–690.

(6) Eastman, P.; Swails, J.; Chodera, J. D.; McGibbon, R. T.; Zhao, Y.; Beauchamp, K. A.; Wang, L. P.; Simmonett, A. C.; Harrigan, M. P.; Stern, C. D.; Wiewiora, R. P.; Brooks, B. R.; Pande, V. S. OpenMM 7: Rapid development of high performance algorithms for molecular dynamics. *PLoS Computational Biology* **2017**, *13*, 1–17.

(7) Donyapour, N.; Roussey, N. M.; Dickson, A. REVO: Resampling of ensembles by variation optimization. *Journal of Chemical Physics* **2019**, *150*, 244112.

(8) Lotz, S. D.; Dickson, A. Wepy: A Flexible Software Framework for Simulating Rare Events with Weighted Ensemble Resampling. *ACS Omega* **2020**, *5*, 31608–31623.

(9) Pedregosa, F. et al. Scikit-learn: Machine Learning in Python. *Journal of Machine Learning Research* **2011**, *12*, 2825–2830.
